# New Forceps for Removing Superior Maxilla

**Published:** 1858-01

**Authors:** 


*New Forceps for Removing Superior Maxilla.*—At a recent meeting of the New York Medical and Surgical Society, Dr. Charles Isaacs exhibited a pair of forceps which he had contrived to facilitate the removal of the superior maxilla. The instrument is a right-angled cutting forceps, and is used in the following manner.

After removing a central and a lateral incisor, an incision is made backwards through the mucous membrane crossing the hard palate a little to the left of the median line, as far as the junction of the palate plate of the upper maxilla with the palatine bone. Here the bone is perforated by a triangular punch with cutting edges, and through this opening is passed into the nasal cavity, a small straight saw, which is made to cut its way outward to the alveolar process, thus severing the connection between the palate plates of the two bones. The superior maxilla is then notched vertically by a small straight saw, near its junction with the malar bone, where it is divided by a single cut of the right-angled forceps. The soft parts having been separated from the floor of the orbit, the nasal process of the superior maxilla and the connection of its orbital plate with the ethmoid are then severed by a straight cutting forceps. One blade of the right angled forceps being then introdued into the nostril and the other into the mouth, the hard palate is divided at the left of the median line by a single clip. One more cut of the right-angled forceps separates the upper jaw from its attachment to the palatine bone, near the pterygoid process of the sphenoid. The hand or a duck-billed forceps grasping the maxilla, thus severed from its connections, removes that bone and completes the operation.

Tieman makes the instrument and sells it under the name of Isaac's right-angled forceps.

